# Membrane targeting of the EF-hand containing calcium-sensing proteins CaBP7 and CaBP8

**DOI:** 10.1016/j.bbrc.2009.01.177

**Published:** 2009-03-20

**Authors:** Hannah V. McCue, Robert D. Burgoyne, Lee P. Haynes

**Affiliations:** The Physiological Laboratory, School of Biomedical Sciences, University of Liverpool, Crown Street, Liverpool, Merseyside L69 3BX, UK

**Keywords:** CaBPs, Calcium, Calcium-binding, Calmodulin, Calneurons, EF-hand, Neurons, Neuro2A cells, Protein targeting

## Abstract

The CaBP family of EF-hand containing small Ca^2+^-binding proteins have recently emerged as important regulators of multiple targets essential to normal neuronal function in the mammalian central nervous system. Of particular interest are CaBP7 and CaBP8, abundantly expressed brain proteins that exhibit the greatest sequence divergence from other family members. In this study, we have analysed their sub-cellular localisations in a model neuronal (Neuro2A) cell line and show that both proteins exhibit a membrane distribution distinct from the other CaBPs and consistent with localisation to the trans-Golgi network (TGN). Furthermore, we show that their localisation to the TGN critically depends upon an unusual predicted C-terminal transmembrane domain that if deleted or disrupted has dramatic consequences for protein targeting. CaBP7 and 8, therefore, possess a targeting mechanism that is unique amongst the CaBPs that may contribute to differential functional Ca^2+^-sensing by these family members.

## Introduction

Calcium (Ca^2+^) signalling impinges on almost all aspects of mammalian physiology [1]. This is well illustrated in the central nervous system (CNS) where Ca^2+^ signals regulate neurotransmission and synaptic plasticity to shape higher level processes such as learning and memory [2,3]. The mammalian CNS is able to utilise a staggering array of complex spatio-temporal Ca^2+^ signals and this is largely attributable to the evolution of families of Ca^2+^-binding proteins that display unique expression patterns and distinct Ca^2+^-sensing properties [4,5].

One such family of Ca^2+^-sensors are the small EF-hand containing Calcium Binding Proteins or CaBPs [5,6]. Members of this family appear structurally related to the ubiquitous EF-hand Ca^2+^-sensing protein calmodulin (CaM) [7], they display, however, restricted expression profiles and respond to specific Ca^2+^ signals. The CaBP family consists of six isoforms (1, 2, 4, 5, 7 and 8) [5,6,8] although alternative splicing of the CaBP1 and possibly CaBP2 genes generates at least nine distinct proteins. Recent research has implicated Caldendrin and its two shorter splice variants, CaBP1-Long and CaBP1-Short, as regulators of Ca^2+^ release channels in mammalian cells. Targets include P/Q [9] and L-Type [10] voltage gated channels of the CNS, N-type channels of neuroendocrine cells [11], intracellular IP_3_-receptors [12,13], TRPC5 channels [14] and Ca_V_1.3 channels in auditory hair cells [15]. Caldendrin is localised to the post synaptic density of excitatory synapses [16] and integrates Ca^2+^ signals from both synaptic and extra-synaptic NMDA receptors to influence synaptic architecture [17]. CaBP4 represents a retinal specific modulator of Ca_V_1.4 channels [18,19] and mutations in the CaBP4 gene are responsible for autosomal recessive night blindness [20]. CaBP5 is also expressed in sensory cell types and in the retina appears to modulate the activity of Ca_V_1.2 channels to perhaps regulate retinal sensitivity [21].

CaBP7 and 8 (also known as calneurons II and I, respectively) are recent additions to the CaBP family and although similar to one another (64% identity) they are most divergent from the other family members sharing ⩽30% identity. Little is known concerning CaBP7 and 8 other than that they are abundantly expressed brain proteins [6,8] that have a unique arrangement of functional and non-functional EF-hand motifs. We have examined the sub-cellular distribution of CaBP7 and 8 and other members of the CaBP family following expression in differentiated Neuro2A cells, a model neuronal cell line. Our results indicate that CaBP7 and 8 share a distinctive membranous localisation and can be co-localised with trans-Golgi network (TGN) specific markers. Interestingly, CaBP7 and 8 lack sequence determinants for lipid modifications seen in some other CaBPs. Analysis of their primary sequences uncovered a predicted C-terminal transmembrane domain (TMD). We have investigated the role of this domain in determining the distinctive sub-cellular distributions of CaBP7 and 8 and provide evidence that it is essential for localisation. Deletion or mutation of the TMD ablates the membrane association of CaBP7 and 8 and furthermore, fusion of the TMDs to the normally cytosolic CaBP5 or mCherry proteins induces localisation to membranes. It has been unclear why so many different CaBPs are expressed but our data provides new insights into the membrane targeting of CaBP7 and 8 that could explain how they may respond to distinct spatially-localised Ca^2+^ signals.

## Materials and methods

*Plasmids constructs.* Full length cDNAs encoding all human CaBPs were obtained by PCR amplification from Quikclone (Clontech, CA, USA) human brain cDNA template. Products were cloned into C-terminally tagging mCherry-N1 or mOrange-N1 vectors (a gift from Dr. R. Tsien, University of California) for expression in mammalian cells. CaBP7 and 8 TMD deleted constructs were generated by PCR cloning from the full length parental vectors. Triple lysine TMD mutant plasmids were generated by site directed mutagenesis (Quikchange, Stratagene, USA) of parental vectors. N-terminally tagged enhanced yellow fluorescent protein (EYFP) variants of CaBP7 and 8 were generated by sub-cloning cDNAs into the EYFP-C1 vector (Clontech, CA, USA). CaBP5 and mCherry-C1 chimaeras containing the CaBP7 or CaBP8 TMDs were generated by PCR amplification of the appropriate TMD and subsequent sub-cloning using standard techniques. CaBP1-Long-EYFP was described previously [12]. The cis/medial Golgi marker (EYFP-Golgi) was obtained from Clontech, CA, USA. All constructs were verified by automated sequencing.

*Cell culture and transfection*. Neuro2A cells were maintained in DMEM supplemented with 10% foetal bovine serum, 1% penicillin/streptomycin and maintained in a humidified atmosphere of 95%air/5% CO_2_ at 37 °C. Prior to transfection, media was exchanged for DMEM containing 2% FBS, 1% penicillin/streptomycin and 20 μM retinoic acid to induce cell differentiation. Cells plated onto glass coverslips were transiently transfected with 1 μg of the appropriate plasmid construct using GeneJuice transfection reagent (Novagen) according to the manufacturer’s protocol. For co-localisation studies cells were transfected with 1 μg of each construct. Cells were fixed 24 h post-transfection in 4% formaldehyde in phosphate buffered saline (PBS) for 30 min. In some cases, cells were processed for immunofluorescence by permeabilisation with PBT (0.1% triton X-100, 0.3% BSA in PBS), incubation with mouse monoclonal anti-syntaxin 6 antibody (Abcam) and subsequent incubation with a FITC conjugated anti-mouse secondary antibody (Sigma).

*VSVG assay*. For cotransfections with VSVG cells were transiently transfected with ts045 VSVG-GFP and each of CaBP7-mCherry, CaBP8-mCherry, mCherry-CaBP7^1–188^, mCherry-CaBP8^1–192^, CaBP7^TMD^ ^KKK^-mCherry, CaBP8^TMD^ ^KKK^-mCherry, CaBP5^CaBP7TMD^-mCherry, CaBP5^CaBP7TMD^-mCherry and mCherry-^CaBP7TMD^. Cells were incubated at 37 °C for 4 h to permit transfection then transferred to 40 °C overnight. Cells were subsequently transferred to 20 °C for 2 h to trap ts045 VSVG-GFP at the TGN before fixation.

*Confocal microscopy*. Fixed cells were imaged using a Leica TCS-SP-MP microscope (Leica Microsystems, Heidelberg, Germany) with a 22 μm pinhole and a ×63 oil immersion objective. Images were exported as TIFF files and compiled in CorelDraw ×4 (Corel Corporation).

## Results

### Localisation of human CaBP isoforms in Neuro2A cells

**Fig. 1:**
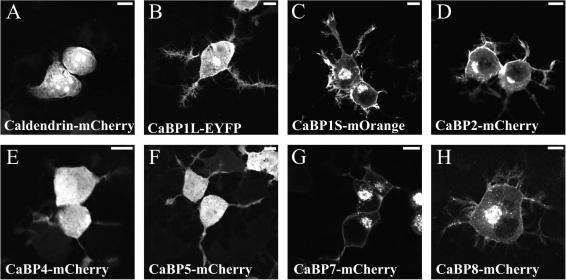
Intracellular distribution of fluorescently tagged human CaBP isoforms. The indicated constructs were used to determine the localisation of CaBPs expressed in retinoic acid differentiated N2A cells. The scale bar represents 10 μm.

In order to compare the sub-cellular localisations of human CaBP isoforms we determined the distributions of each of the proteins following transfection of colour tagged constructs into retinoic acid differentiated neuro2A (N2A) cells [22]. As the CaBPs exhibit greatest expression levels in neuronal tissues we reasoned that these cells would provide a more accurate reflection of correct sub-cellular localisation. CaBP1-Long, CaBP1-Short and CaBP2 all harbour an N-terminal myristoylation consensus sequence and our results show that these isoforms were correctly trafficked and efficiently membrane associated, localising to both a peri-nuclear region resembling the Golgi apparatus and the plasma membrane (Fig. 1B–D). Of the remaining CaBPs, none carry consensus sequences for post-translational lipidation and caldendrin, CaBP4 and CaBP5 (Fig. 1A, E and F) all exhibited a predominantly diffuse cytosolic distribution consistent with this. CaBP7 and 8 similarly lack consensus motifs for post-translational acylation but both proteins were membrane associated in N2A cells (Fig. 1G and H). CaBP7 and 8 distributions were similar, exhibiting localisation predominantly to a peri-nuclear compartment, intracellular vesicles and in some instances the plasma membrane. This unexpected localisation led us to investigate the cellular structures labelled by CaBPs7 and 8 and the precise mechanism of this membrane association further.

### Association of CaBP7 and 8 with the TGN

**Fig. 2:**
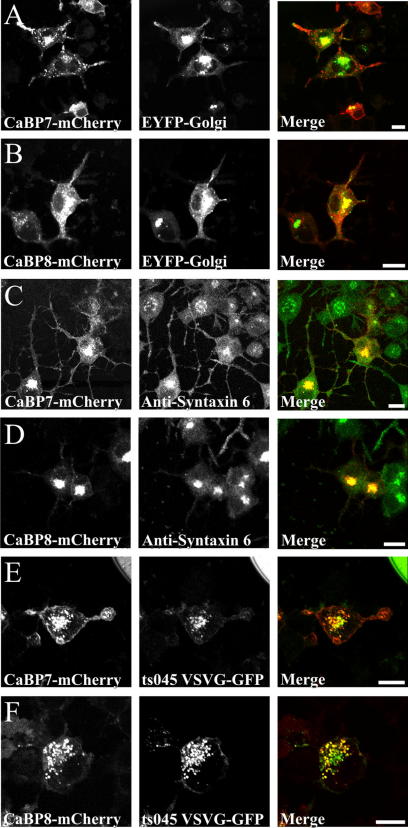
Co-localisation of CaBP7 and 8 with TGN markers in differentiated N2A cells. Localisation of CaBP7 and 8 (red) with an EYFP tagged cis/medial Golgi marker (A,B, green) or the TGN specific markers syntaxin 6 (C,D, green) and ts045 VSVG-GFP (E,F, green) was examined. Regions of co-localisation appear yellow in the merged images. The scale bar represents 10 μm. (For interpretation of color mentioned in this figure the reader is referred to the web version of the article.)

We initially investigated the identity of the peri-nuclear compartment labelled by CaBP7 and 8 in differentiated N2A cells by utilising a cis/medial Golgi marker (Fig. 2A and B). We observed only partial co-localisation in these analyses and since previous studies had determined a TGN localisation for CaBP1-Long and CaBP1-Short we proceeded to examine whether the peri-nuclear compartment labelled by CaBPs7 and 8 correlated more extensively with this Golgi sub-domain. We utilised two TGN specific markers, syntaxin 6 [23] and the constitutive secretory cargo, vesicular stomatitis virus-G protein (VSVG) [24] to determine the extent of co-localisation in cells co-expressing CaBP7 or 8. Initially, we examined the distributions of CaBPs7 and 8 with endogenous, immunolabelled, syntaxin 6 (Fig. 2C and D) and observed co-localisation consistent with the presence of both proteins at the TGN. In examining co-localisation with VSVG we exploited a temperature sensitive green fluorescent protein tagged version of the protein (ts045 VSVG-GFP) [25] that has a temperature dependent reversible folding defect permitting trapping of the protein in the endoplasmic reticulum (ER). Cells were maintained at 40 °C post-transfection (to trap ts045 VSVG-GFP in the ER) then shifted to 20 °C to allow traffic from the ER to the TGN (here transport is again blocked as vesicular traffic out of the TGN is inhibited at 20 °C). Under these conditions both CaBP7 and 8 co-localised extensively with the ts045 VSVG-GFP positive TGN compartment (Fig. 2E and F) consistent with syntaxin 6 data.

### Identification and analysis of putative C-terminal TMDs in CaBPs7 and 8

We examined the primary sequence of CaBP7 and 8 to search for determinants that might be responsible for sub-cellular targeting. The TMpred algorithm (http://www.ch.embnet.org/software/TMPRED_form.html) [26] strongly predicted the presence of a C-terminal TMD in both CaBP7 (residues 189–205) and CaBP8 (residues 193–209). Of the other CaBP family members only CaBP1-Long and CaBP2 were predicted to contain a TMD although prediction scores in these instances were significantly lower than those for CaBP7 and 8. Since for CaBP1-Long N-terminal myristoylation has been proven essential to membrane association of the protein [12] we focused upon investigating the predicted TMDs in CaBPs7 and 8 and their potential role in sub-cellular localisation.

### Characterisation of CaBP7 and 8 C-terminal TMDs

**Fig. 3:**
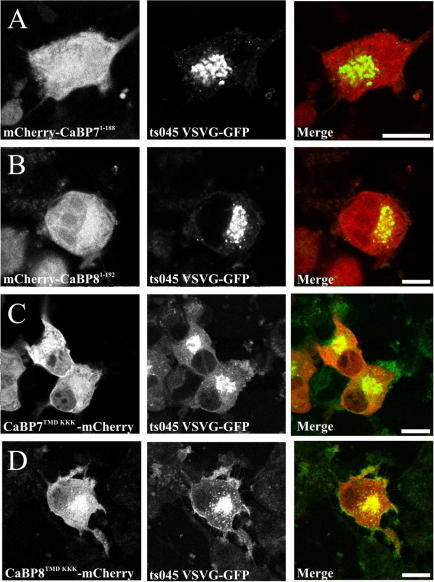
Analysis of the function of the putative TMD of CaBP7 and 8 TMD in differentiated N2A cells. Cells were transfected with ts045 VSVG-GFP (green) as a TGN marker and either CaBP7^1–188^ or CaBP8^1–192^ TMD deletion mutants (A,B, red); CaBP7^TMD–KKK^ or CaBP8^TMD–KKK^ TMD triple lysine mutants (C,D, red). Co-localisation appears yellow in merged images. The scale bar represents 10 μm. (For interpretation of color mentioned in this figure the reader is referred to the web version of the article.)

In order to provide evidence that predicted C-terminal TMDs in CaBPs7 and 8 were of functional relevance we first generated truncation variants of each protein terminating at the amino acid directly before the beginning of the predicted TMD. These constructs (CaBP7^1–188^ or CaBP8^1–192^, Fig. 3A and B) were predominantly cytosolic in contrast to the defined membrane localisation of the wild-type proteins (Fig. 1G and H) and co-localisation with the TGN was lost. In related studies, we utilised a mutagenesis approach to replace the central three amino acids of the TMDs in full-length CaBPs7 and 8 with lysine residues (predicted by TMpred to abolish TMD function). In comparison to the localisation observed with full-length wild-type CaBP7 and 8 these mutants (CaBP7^TMD–KKK^ and CaBP8^TMD–KKK^, Fig. 3C and D) exhibited an increased cytosolic distribution that was more pronounced for CaBP7. These data are consistent with those obtained for truncation constructs and suggest that disruption of the TMDs of CaBP7 or CaBP8 leads to extensive mislocalisation of each protein into the cytosol.

### Analysis of CaBP7 and 8 TMD chimaeric constructs

**Fig. 4:**
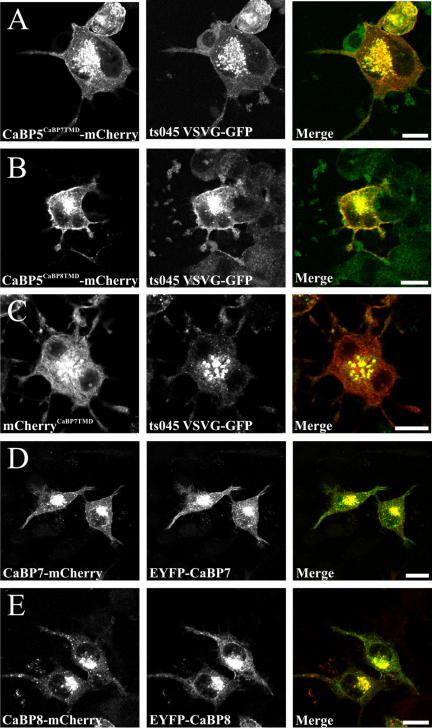
Analysis of CaBP5 and mCherry chimaeric constructs in retinoic acid differentiated N2A cells and effect of the position of the colour tag in CaBP7 and 8. The figure shows CaBP5^CaBP7TMD^ or CaBP5^CaBP8TMD^ chimaeras (A,B, red), or mCherry^CaBP7TMD^ (C, red) co-expressed with ts045 VSVG-GFP (green) as a TGN marker. C-terminally mCherry tagged CaBP7 and 8 (red) were co-expressed with N-terminally EYFP tagged variants of the same proteins (green, D,E). Regions of co-localisation appear yellow in merged images. The scale bar represents 10 μm. (For interpretation of color mentioned in this figure the reader is referred to the web version of the article.)

In an extension to our initial characterisation studies we generated chimaeric constructs fusing each TMD sequence to the C-terminus of CaBP5 or mCherry both of which are normally cytosolic proteins. In comparison to the normal localisation of wild-type CaBP5 (Fig. 1F) both the CaBP5^CaBP7TMD^ and CaBP5^CaBP8TMD^ fusion constructs (Fig. 4A and B) co-localised extensively with the TGN marker ts045 VSVG-GFP. Similarly, the TMD of CaBP7 when fused to the fluorescent protein mCherry (Fig. 4C) was also able to direct a pool of this protein to the TGN. These observations further support the notion that the putative TMDs of CaBPs7 and 8 are necessary and sufficient for targeting to specific sub-cellular membrane domains. As all of our data indicated the presence of a C-terminal membrane interaction motif in CaBP7 and 8 we decided to rule out the possibility that fusing a fluorescent protein tag to this end of the molecule affects targeting. To this end we re-cloned CaBP7 and 8 into an N-terminally tagging fluorescent vector and co-expressed these constructs with their C-terminally tagged counterparts in the same cells. Complete co-localisation was observed in these experiments indicating that tag position did not influence the sub-cellular distribution of CaBP7 or 8 (Fig. 4D and E).

## Discussion

The CaBP family of EF-hand containing small Ca^2+^-binding proteins have emerged as important regulators of a variety of plasma membrane and intracellular cation channels in neuronal cell types principally modulating cytosolic Ca^2+^ concentrations through control of influx from the extracellular fluid or efflux from intracellular stores. The need for multiple members of this family is not yet clear. Based on the documented functions exerted by CaBPs1, 4 and 5, we reasoned it likely that other family members would possess important, potentially neuronal specific, activities. In this study, we have characterised CaBP7 and 8 the two most divergent members of this family. These proteins are expressed to high levels in distinct regions of the adult mammalian brain and display developmental changes in expression consistent with a role in normal neuronal function. The only report published to date describing an analysis of endogenous CaBP7 and 8 localisation in primary hippocampal cultures [6] was based on crude subcelluar fractionation and concluded that both proteins were present in the soluble cell fraction suggesting that these proteins, like some other members of the CaBP family are normally cytosolic. This finding was consistent with a lack of consensus motifs in CaBPs7 and 8 for post-translational acylation, the only modification to date that has been identified in the CaBP family to mediate membrane association. In comparative studies examining all human CaBP isoforms expressed in N2A cells we observed a distinctive mechanism for membrane localisation for CaBPs7 and 8. All other CaBP family members in this analysis exhibited localisation patterns as previously reported and/or consistent with the presence or absence of intrinsic myristoylation consensus sequences that can fully account for the membrane localisation of CaBP1-Long, CaBP1-Short and CaBP2 [5,12,13].

Co-localisation studies showed that CaBP7 and 8 were targeted to and associated with the TGN and other membranous structures in neuronally-differentiated N2A cells. This was based on extensive co-localisation of CaBP7 and 8 with the membranous TGN marker syntaxin 6 and the secretory cargo protein ts045 VSVG-GFP in a well characterised protocol that was designed to trap it in the TGN. Interrogation of the CaBP7 and 8 primary sequences using the TMpred algorithm uncovered a putative C-terminal TMD in both proteins with a high probability score and which closely resembled known TMDs present in tail anchored proteins [27]. To test the physiological relevance of the predicted TMD for membrane targeting we deleted the domain entirely from each protein and observed the resulting effect on localisation. In N2A cells expressing truncated versions of CaBPs7 and 8 we observed a complete loss of membrane localisation and the proteins were entirely cytosolic. Removing the C-terminal portion of the protein could potentially affect protein folding leading to mislocalisation and therefore we tested the function of the TMD in independent studies. Generation of triple lysine mutants predicted to completely disrupt the putative TMD allowed us to examine TMD function in the full-length proteins. In agreement with our truncation construct data, the lysine mutants associated with cellular membranes less efficiently and an increase in cytosolic signal was observed. This effect was most severe in the case of CaBP7^TMDKKK^ whilst CaBP8^TMDKKK^ retained a significant level of membrane association. It is possible that sequences flanking the TMD of CaBP8 are responsible for mediating residual membrane attachment in the presence of a mutated TMD. Finally we examined whether the putative TMDs of CaBP7 and 8 could direct a related yet normally cytosolic protein to membrane domains if attached at the C-terminus. Using CaBP5 and mCherry as test proteins and fusing to them the TMD of either CaBP7 or 8 we were able to generate chimaera’s that efficiently targeted to sub-cellular compartments similar in appearance to those observed with wild-type CaBP7 and 8. Cumulatively these data argue that both CaBPs7 and 8 contain fully functional C-terminal TMDs in common with other tail-anchored proteins and more importantly that these regions are necessary and sufficient in driving membrane localisation. Since our expression constructs were all C-terminally tagged (which could interfere with the function of a C-terminal TMD) we confirmed that moving the tag to the N-terminus of CaBP7 and 8 did not alter the observed sub-cellular distributions of the proteins.

In this study, we have begun dissecting the biochemical properties of CaBP7 and 8, Ca^2+^-sensing proteins highly expressed in adult mammalian brain. We have identified these proteins as having a distinct sub-cellular distribution pattern resulting from the presence of a predicted C-terminal TMD homologous to those of tail-anchored proteins. This is intriguing as no other characterised CaBPs have been found to utilise such a mechanism as a means of membrane association. The prototypical EF-hand containing Ca^2+^-sensor CaM is a cytosolic protein that can become transiently membrane associated on interaction with membrane bound effectors in response to Ca^2+^. Evolutionary more recent additions to the CaM superfamily including the NCS proteins [4] and the CaBPs [5,6] have evolved more direct mechanisms to associate with target membranes based around post-translational myristoylation, a modification that itself has been extensively elaborated upon to permit permanent or transient membrane association [28]. The introduction of a C-terminal tail-anchor into the CaBP family is likely to be part of the evolution in targeting complexity observed in the CaM superfamily. In the case of CaBPs7 and 8 this would permit the sensing of spatially restricted, specialised, intracellular Ca^2+^ signals particularly in the region of the TGN.
